# All-cause Mortality Due to Bacteremia during a 60-Day Non-Physician Healthcare Worker Strike

**DOI:** 10.1093/cid/ciaa1373

**Published:** 2020-09-12

**Authors:** Filip Jansåker, Mona Katrine Alberthe Holm, Kim Oren Gradel, Jenny Dahl Knudsen, Jonas Bredtoft Boel, Danish Collaborative Bacteraemia Network (DACOBAN), J D Østergaard, U S Knudsen, M Jensen, M Arpi, S Thønnings Pinholt, H C Schønheyder, M Søgaard, K Koch, J Smit, K O Gradel

**Affiliations:** 1 Department of Clinical Microbiology, Rigshospitalet, Copenhagen, Denmark; 2 Center for Primary Health Care Research, Lund University, Malmö, Sweden; 3 Department of Clinical Microbiology, Hvidovre Hospital, Hvidovre, Denmark; 4 Center for Clinical Epidemiology, Odense University Hospital, Odense, Denmark; 5 Research Unit of Clinical Epidemiology, Department of Clinical Research, University of Southern Denmark, Odense, Denmark; 6 Department of Clinical Microbiology, Herlev University Hospital, Herlev, Denmark

**Keywords:** healthcare strike, bacteremia, bloodstream infection, mortality, epidemiology

## Abstract

This study explored all-cause mortality of bacteremia diagnosed during a 60-day non-physician healthcare worker strike in 2008. A significant change, with 5.0% (95% confidence interval [CI] 1.2–8.7%, *P* < .01) absolute risk increase, was seen in 90-day mortality during the strike (n = 598) compared with the rest of the study period 2000–2015 (n = 75 647).

Strikes by physicians or non-physician healthcare workers (HCWs) have been a global occurrence for several decades, and it has been debated whether they are ethical or morally justifiable due to the potential increase of mortality in patients [1]. Even though HCW strikes obviously often have an impact on the healthcare services, most studies have not found any increase in mortality associated with HCW strikes, especially when emergency services have been maintained [2–5]. A paradoxical pattern of decreased mortality during strikes by physicians, followed by increased mortality thereafter, has in some cases been attributed to resumption of elective surgeries [3, 6].

Few studies have investigated the association between non-physician HCW strikes and mortality [4, 5, 7], and to our knowledge none have specifically studied patients with bacteremia: an acute and severe infection is a frequent and leading cause of death and contributes to a large healthcare burden [8]. Affected patients often require a considerable amount of healthcare to be managed properly, including early diagnosis and treatment. This population of patients may thus be feasibly susceptible to even slight decreases in healthcare services.

In 2008, the majority of the Danish nurses and other non-physician HCWs went on a 60-day strike due to a national conflict [9]. The healthcare services were available on a decreased level during that period. The aim of this study was to investigate the all-cause mortality in patients diagnosed with bacteremia during the strike period compared to the nonstrike period.

## MATERIALS AND METHODS

### Study Setting

The design was a retrospective population-based cohort study comprised of patients with bacteremia from 2 of Denmark’s 5 regions. The involved patients were from The Capital Region and The North Denmark Region, representing 42% of Denmark’s approximately 5.7 million inhabitants. The cases were identified through the departments of clinical microbiology serving this population: Aalborg University Hospital, Herlev University Hospital, Hvidovre University Hospital, and Rigshospitalet. All Danish residents have a unique 10-digit civil registration number (CPR), which is used for linkage between high quality healthcare and other types of registries with potential for population-based research.

### Strike

The conditions of the 2008 strike were summarized by Kronberg et al [10], but in short, in most wards, the majority of nurses and other non-physician HCWs went on strike from April 16th to June 14th, 2008, due to a national conflict with the employer [9]. Thus, a minimum number of nursing staff securing emergency care was on duty during the strike and over 370 000 surgical procedures were cancelled. The strike did not affect wards of intensive care, oncology, or pediatrics.

### DACOBAN

The data were collected through a research database containing microbiological data for all positive blood cultures from the departments of clinical microbiology: DACOBAN. The data were collected from 1 January, 2000 through 29 October, 2015. All cases of pathogenic bacteria were registered, but contaminants such as, inter alia, *Cutibacterium acnes*, coagulase-negative staphylococci, *Corynebacterium spp., Bacillus spp.* were excluded from the database. But, if two isolates of the contamination species were found within 5 days from the same patient, the findings were registered as bacteremia. The DACOBAN contains data from 3 sources:

1) The Clinical Microbiological Laboratory Information Systems AdBact (Autonic AB, Sweden) and MADS (Department of Clinical Microbiology, Aarhus University Hospital, Denmark).
2) The Danish National Patient Registry (DNPR): data on hospital admission and discharge; diagnosis (International Classification of Diseases, ICD-10 codes, revision 10), enabling us to calculate the Charlson Comorbidity Index (CCI) based on the first-time Charlson disease diagnosis prior to the bacteremia episode.
3) The Danish Civil Registration System (CRS): data on vital status registered as alive, dead, or emigrated).

### Approval and Ethics

The use of the DACOBAN database was approved according to the guidelines of the Regional Committee on Health Research Ethics for use of clinical and laboratory data (Danish Data Protection Agency, record 2007-41-0627). For this study, all data were made anonymous prior to the analysis.

### Statistical Analyses

The study outcome was death from any cause within 90 days after the bacteremia episode. The mortality rate of patients with bacteremia was compared between the strike period and the rest of the study period. Cox regression analyses were used to compare the mortality rates between the 2 groups adjusting for year of infection, month of infection, age, sex (male or female), acquisition of infection (community, hospital associated, or nosocomial), mono- or polymicrobial infection, and Charlson Comorbidity Index (low [0], medium [1–2], or high [>2] index score). We tested the model for proportionality using Schoenfeld residuals and performed a time-split analysis (for day 0–30 and day 31–90) to identify possible changes in mortality, respectively. For all-cause 90-day mortality a *post hoc* analysis on absolute respectively relative risk difference was conducted (alpha 0.05). All analyses were done in R version 3.6.3.

## RESULTS

Table 1 includes patient characteristics and all-cause mortality. The study included 76 245 cases of bacteremia, of which 598 occurred during the 60-day strike period in 2008.

**Table 1.** Patient Characteristics and All-cause Mortality in Patients Diagnosed With Bacteremia During the Non-Physician Healthcare Personnel Strike in Denmark^a^

	Non-strike (n = 75 647)	Strike (n = 598)	Total (n = 76 245)	*P* value
Sex				.58
Female	34 322 (45.4%)	278 (46.5%)	34 600 (45.4%)	
Male	41 325 (54.6%)	320 (53.5%)	41 645 (54.6%)	
Age				.55
Median	70.6	70.4	70.6	
IQR	57.9, 80.8	59.7, 81.0	57.9, 80.8	
Origin of infection				.32
Community-acquired	34 580 (45.7%)	286 (47.8%)	34 866 (45.7%)	
Healthcare related	18 014 (23.8%)	138 (23.1%)	18 152 (23.8%)	
Nosocomial	23 053 (30.5%)	174 (29.1%)	23 227 (30.5%)	
Charlson Comorbidity Index				.89
Low (0)	19 710 (26.1%)	143 (23.9%)	19 853 (26.0%)	
Medium (1–2)	27 336 (36.1%)	239 (40.0%)	27 575 (36.2%)	
High (>2)	28 601 (37.8%)	216 (36.1%)	28 817 (37.8%)	
Polymicrobial infection	7 536 (10.0%)	61 (10.2%)	7 597 (10.0%)	.85
Crude all-cause mortality rate				
0–30-day mortality	15 141 (20.0%)	135 (22.6%)	15 276 (20.0%)	.12
31–90-day mortality	6 515 (8.6%)	66 (11%)	6518 (8.6%)	.04
0–90-day mortality	21 656 (28.6%)	201 (33.6%)	21 857 (28.7%)	<.01
Crude hazard ratio (95% CI)				
0–30-day mortality	Ref.	1.16 (.97 to 1.37)		.21
31–90-day mortality	Ref.	1.32 (1.01 to 1.73)		.04
0–90-day mortality	Ref.	1.20 (1.04 to 1.39)		.01
Adjusted hazard ratio^b^ (95% CI)				
0–30-day mortality	Ref.	1.20 (1.00 to 1.42)		.05
31–90-day mortality	Ref.	1.39 (1.07 to 1.81)		.01
0-90-day mortality	Ref.	1.25 (1.08 to 1.44)		<.01

Abbreviations: CI, confidence interval; CCI, Charlson Comorbidity Index.

^a^From 16 April to 14 June 2008, compared to the rest of the period 2000–2015.

^b^Adjusted for month, year, sex, age, acquisition of infection, polymicrobial infection, and CCI.

No significant difference in patient characteristics was seen between the 2 groups. We identified a significant difference in the 90-day all-cause mortality, with a significantly higher mortality associated with the strike period compared to the rest of the period [ie, 33.6% versus 28.6% (*P* < .01)]. This corresponds with a 17.4% (95% CI 4.8-31.5%, *P* < .01) increase of the relative risk and 5.0% (95% CI 1.2–8.7%, *P* < .01) increase of the absolute risk.

The crude hazard ratio (HR) for all-cause mortality, when comparing the strike period to the rest of study period, was significantly higher for 31–90-day and 0–90-day, respectively, but not for 0–30-day. When adjusting for covariates in these analyses, the hazard ratios increased for all periods.

Supplementary material Figure 1 shows the 30-day and 90-day mortality rates from 2000 through 2015.

## DISCUSSION

To our knowledge, this is the first study that investigates the changes in mortality during a non-physician healthcare worker strike in patients with bacteremia. We found that increased mortality, especially beyond 30 days, could be associated with the strike.

The significantly increased risk of 90-day mortality associated with the strike could be explained due to the increase in the 31–90-day mortality (HR 1.39, 95% CI 1.07 to 1.81, *P* = .01). However, when adjusting for relevant covariates (month, year, sex, age, acquisition of infection, polymicrobial infection, and CCI) the weaker associated increased risk in the 0–30-day mortality to the strike period was almost significant (HR 1.20, 95% CI 1.00 to 1.42, *P* = .05).

In addition, there was a general trend of decreased mortality of bacteremia diagnosed during the period April 16 to June 14 over the observed 15 years (Supplementary material Figure 1), especially after 2008. In this figure, an increase in 90-day all-cause crude mortality during the strike is viewed, but not for 30-day all-cause crude mortality.

The only other study that we are aware of investigating healthcare-related outcomes of the 2008 strike in Denmark found a significant short-term decrease of “care around birth” (eg, midwife and nurse consultations) during the strike period, with consequently more contacts to general practitioners [10].

Other potential reasons for the associated increased mortality observed in patients diagnosed with bacteremia during the strike period could, in addition to the minimum number of HCWs during the strike period, potentially also be associated with the likely resumption of elective procedures or resumption to duty of less experienced HCWs after the strike, as reported by other studies [3, 6].

In general, studies on the change in mortality during healthcare worker strikes have not shown an increase of mortality associated with the strikes [2–5], except in isolated cases, compromised of mainly physician strikes, where indications of increased mortality have been suggested [2, 11, 12], particularly in children [2, 12]. To our knowledge, few studies have investigated any change in mortality associated with non-physician HCW strikes [4, 5, 7], of which only one study described an increase in mortality during a nurse strike [7]. Moreover, previous studies do not seem to have singled out patients with bacteremia, who generally are dependent on extensive healthcare services. Other strengths of our study were its validated databases and the inclusion of severe comorbidities, acquisition of infection, and microbiological data. The strike, and its effect on healthcare services, lasted over a fairly long time period; and the study period was conducted over 15 years, which made it possible to include a large control group.

An important limitation of our database is the lack of data for the severity of the acute illness, and as an observational study a cause and effect relationship cannot be established from these data. Likewise, we cannot tell whether the increased mortality occurred in the specific wards where fewest nurses were present during the strike. In conclusion, a significant increase in all-cause 90-day mortality was found in patients diagnosed with bacteremia during the 60-day non-physician healthcare worker strike compared to the rest of the period 2000–2015. Although the present study does not prove causality, it is possible that the strike contributed to an increased mortality in this particular group of patients. The findings indicate that vulnerable groups of patients need extra attention during strikes.

## Supplementary Data

Supplementary materials are available at Clinical Infectious Diseases online. Consisting of data provided by the authors to benefit the reader, the posted materials are not copyedited and are the sole responsibility of the authors, so questions or comments should be addressed to the corresponding author.

## Supplementary Material

ciaa1373_suppl_Supplementary_Material

## Notes

***Author contributions.*** All authors take responsibility for the integrity of the data and the accuracy of the data analyses and have approved the final version of the manuscript. Concept: J. D. K. Development of idea: F. J. and J. D. K. Design: all authors. Access, acquisition, and analysis of data: all authors. Statistical analysis: J. B. B., supervision from K. O. G. Interpretation of data: all authors. Drafting of manuscript: F. J. and J. B. B. Critical revision of the manuscript for intellectual content: J. D. K., K. O. G., and M. K. A. H. Guarantors and supervision: J. D. K. and K. O. G. The authors attest that all listed authors meet authorship criteria and that no others meeting the criteria have been omitted.

***Acknowledgments.*** The authors thank the DACOBAN network for making this study possible. Contributing members of DACOBAN include C. Østergaard, J. D. Knudsen, U. S. Jensen, M. Arpi, M. Pinholt, S. Thønnings (Copenhagen, Denmark); H. C. Schønheyder, M. Søgaard, K. Koch, J. Smit (Aalborg, Denmark); and K. O. Gradel (Odense, Denmark).

***Potential conflicts of interest.*** The authors: No reported conflicts of interest. All authors have submitted the ICMJE Form for Disclosure of Potential Conflicts of Interest.
