# Supplementary material for: All-cause Mortality Due to Bacteremia during a 60-Day Non-Physician Healthcare Worker Strike
Source: Clin Infect Dis. 2020 Sep 12;73(7):e1758–61. doi: 10.1093/cid/ciaa1373 (PMC8852811; doi:10.1093/cid/ciaa1373)

**Supplementary material**

**Figure 1.** Trend of all-cause crude 30-day and 90-day (dashed line) mortality in patients with bacteraemia diagnosed from April 16^th^ to June 14^th^ in the years of 2000 to 2015 (black dot highlights strike period 2008)


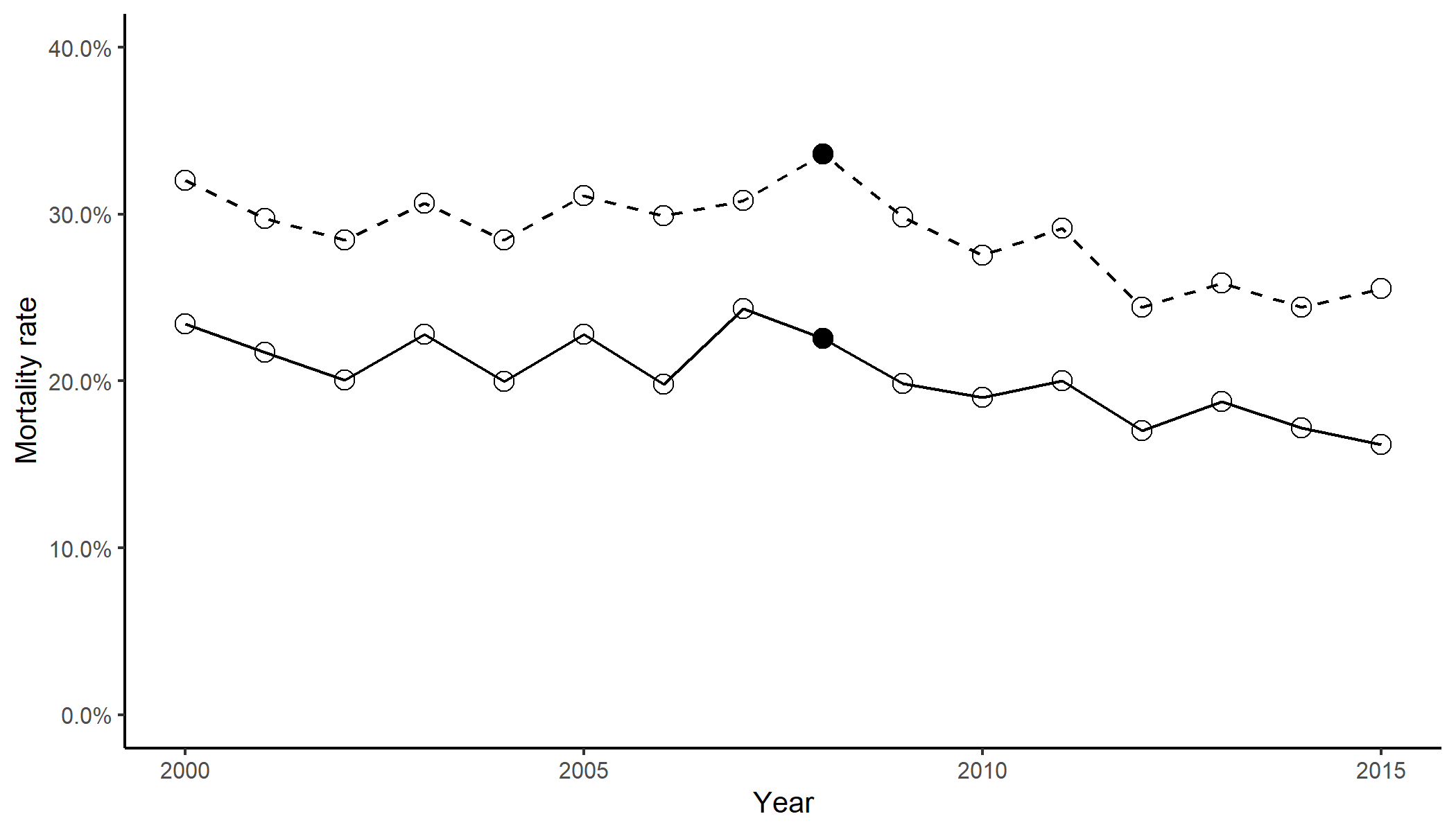

Supplement: ciaa1373_suppl_Supplementary_Material [file ciaa1373_suppl_supplementary_material.docx]
